# A nomogram for assessing secondary infection risk in hospitalized patients with intestinal CRE colonization: a prospective cohort study

**DOI:** 10.1186/s12941-026-00867-2

**Published:** 2026-05-29

**Authors:** Xiaoying Xie, Lijia Ni, Guanping Chen, Jiajian Guo, Xinlu Dai, Mingming Chen, Zhaofan Luo

**Affiliations:** 1 Department of Clinical Laboratory, The Seventh Affiliated Hospital of Sun Yat-sen University, 628 Zhenyuan Road, Shenzhen, 518107 China; 2 Department of Clinical Laboratory, Sun Yat-sen Memorial Hospital, Sun Yat-sen University, Guangzhou, 510120 China

**Keywords:** Carbapenem-resistant *Enterobacterales* (CRE), Intestinal colonization, Secondary infection, Risk prediction model, Whole-genome sequencing

## Abstract

**Objective:**

To investigate the epidemiology, risk factors, and molecular characteristics of intestinal carbapenem-resistant *Enterobacterales* (CRE) colonization and subsequent secondary infections in a hospital setting, and to develop a predictive model for risk stratification.

**Methods:**

A large-scale, unbiased active screening for intestinal CRE carriage was conducted among patients (including physical examination, outpatient, and inpatient populations) between August 31, 2021, and February 28, 2022. A prospective cohort design was used to follow colonized inpatients for six months. Clinical data were collected for univariate and multivariate logistic regression analysis to identify independent risk factors for infection. A nomogram prediction model was constructed and validated. Homology between colonizing and infecting isolates was assessed using WGS and MALDI-TOF MS analysis.

**Results:**

The overall CRE colonization rate was 4.95% (475/9,615), with significant variation between inpatients (5.55%), outpatients (3.97%), and physical examination subjects (3.54%). *Escherichia coli* (49.05%) and *Klebsiella pneumoniae* (46.95%) were the dominant species. The overall secondary infection rate among colonized inpatients was 5.04% (18/357), with the highest rates observed in the Rehabilitation (30.00%), ICU (21.88%), and Neurology (20.00%) departments. Multivariate analysis identified ICU stay ≥ 48 h, carbapenem exposure, fecal incontinence, and tracheostomy as independent risk factors, while admission to Rehabilitation was a protective factor. The prediction model demonstrated high discriminative ability (AUC = 0.960) in this single-center derivation cohort, but external validation is required before clinical application. Molecular analysis revealed that ST11 CRKP was the predominant clone (70%), and most infections (12/18) were genetically homologous to the patient’s colonizing strain.

**Conclusion:**

CRE intestinal colonization is prevalent across healthcare settings. The progression from colonization to infection is driven by specific clinical risks, which can be used to assess risk by our novel nomogram. These findings underscore the need for targeted screening in high-risk departments, enhanced antimicrobial stewardship, and focused surveillance on dominant clones like ST11 CRKP to effectively prevent CRE transmission and infection.

**Supplementary Information:**

The online version contains supplementary material available at 10.1186/s12941-026-00867-2.

## Introduction

Carbapenem-resistant *Enterobacterales* (CRE) pose a catastrophic threat to public health, causing infections that are exceedingly difficult to treat with existing antibiotics and are associated with alarmingly high mortality rates [1]. In response to this crisis, the World Health Organization (WHO) has designated CRE as one of its priority pathogens, underscoring the urgent need for enhanced surveillance and prevention strategies [2]. The resistance of CRE is primarily mediated by the production of carbapenemase enzymes, though other mechanisms, such as efflux pump mutations combined with porin deficiencies, also contribute [3]. This pan-drug resistance, coupled with the capacity for clonal transmission and horizontal gene transfer, makes CRE a formidable challenge in healthcare settings worldwide [4].

The clinical management of CRE infections remains severely limited, often depending on less effective or more toxic second-line antibiotics such as polymyxins, tigecycline, and fosfomycin [5]. Despite rigorous infection control measures, CRE continues to spread within hospitals, underscoring the need for more targeted strategies [6]. A key to its pathogenesis and dissemination lies in gastrointestinal colonization, where the intestinal tract acts as the primary reservoir [7]. Asymptomatic carriers form a silent reservoir, facilitating nosocomial transmission through the fecal-oral route and environmental contamination. Therefore, elucidating the epidemiology of CRE carriage, especially among high-risk inpatients (e.g., in ICUs, pediatric, and hematology wards), is crucial for interrupting transmission chains [8–11].

The progression from silent intestinal colonization to active infection marks a critical and dangerous transition in clinical care. Colonization is a major risk factor for secondary infection, particularly in immunocompromised hosts or patients undergoing invasive procedures [12]. CRE can translocate across the damaged intestinal mucosa, leading to life-threatening conditions including bloodstream infection (BSI), pneumonia, and intra-abdominal infections [13–16]. Approximately 16.5% of colonized patients progress to systemic infection, with risk substantially higher in vulnerable groups such as those with hematological malignancies or transplant recipients [17–19]. This progression is driven by a combination of host and iatrogenic factors, including prior antibiotic exposure [20], comorbidities such as renal disease [21], granulocytosis, and invasive device use [21, 22]. Consequently, CRE infections are independently associated with significantly increased morbidity, mortality, and healthcare costs [23, 24].

Despite the clear and present danger, current clinical practice lacks robust tools to predict which colonized patients are at the highest risk of developing a secondary infection. Existing models often suffer from inadequate specificity and fail to incorporate dynamic risk factors, leaving clinicians without reliable means for preemptive intervention [25]. While decolonization strategies have been explored, their efficacy is inconsistent and they are not routinely recommended due to concerns about promoting further resistance [26]. Therefore, the current paradigm advocates for targeted infection control based on individualized risk assessment.

To address this critical gap, we conducted a prospective cohort study to investigate the clinical and microbial determinants of secondary infections in CRE-colonized individuals. This study was initiated with extensive, unbiased active screening for intestinal CRE carriage, followed by a six-month follow-up period to monitor for secondary infection. Within this cohort framework, we first performed a retrospective analysis to identify key risk factors. These factors were then integrated to construct a nomogram-driven predictive model, aiming to facilitate personalized clinical decision-making. Furthermore, to elucidate the pathogen dynamics underpinning these infections, we employed whole-genome sequencing (WGS) and homology analysis to identify the predominant strains and transmission patterns. By bridging clinical epidemiology with genomic insights, this work aims to enhance our understanding of CRE infection pathways and provide an actionable tool for the early prediction and prevention of secondary infections in CRE-colonized patients.

## Materials and methods

### Study design

We conducted a large-scale, prospective cohort study involving unbiased active screening for intestinal CRE carriage. The study enrolled patients who attended a comprehensive tertiary care hospital in Guangzhou, China, between August 31, 2021, and February 28, 2022. CRE-colonized inpatients were subsequently monitored for a six-month period for the occurrence of secondary infection. The study protocol was approved by the Institutional Review Board (approval number: 2023-KY-117), and informed consent was obtained from all participants.

### Participants, study groups and definitions

During the initial screening phase for intestinal CRE colonization, participants were enrolled consecutively without stratification. For subsequent analysis, the participants were categorized into three groups: (1) Physical examination subjects: Individuals visiting the hospital’s health management center for routine, self-paid, or employer-sponsored health check-ups, who were not seeking care for a specific acute or chronic illness. (2) Outpatients: Patients visiting specialist clinics for consultation, diagnosis, or minor procedures without requiring an overnight hospital stay. (3) Inpatients: Patients formally admitted to a hospital ward for at least one night. Those with positive CRE screening results were further stratified based on clinical outcome into two groups: a “Secondary infection group”, comprising patients who developed a confirmed CRE infection following colonization, and a “Colonized uninfected group”, consisting of those who remained free of CRE infection during follow-up.

CRE colonization was defined as the detection of CRE in fecal specimens in the absence of clinical signs or symptoms of active infection. CRE infection was defined according to combined microbiological and clinical criteria. The isolation of CRE from normally sterile sites (e.g., blood or cerebrospinal fluid) was considered definitive evidence of infection. When CRE was isolated from non-sterile sites (e.g., respiratory tract, urine, wounds), a diagnosis of infection required the presence of compatible clinical features, such as: (1) systemic or local signs (e.g., fever > 38 °C, cough with purulent sputum, pulmonary rales, dysuria, or wound erythema/swelling/purulence); (2) significant microbiological growth, defined as sputum (≥ 10⁶ CFU/mL), lower respiratory secretions from bronchoscopy (≥ 10⁵ CFU/mL), bronchoalveolar lavage fluid (≥ 10⁴ CFU/mL), clean-catch urine (≥ 10⁵ CFU/mL), or any growth from blood/sterile fluids; and (3) supporting radiological evidence (e.g., new pulmonary infiltrates on chest X-ray).

For tracking purposes, each patient with a confirmed CRE infection was assigned a unique identification number starting with “1”. The corresponding infecting isolate was labeled with the suffix “01”, and the colonizing isolate (if available) with “02”. For example, for patient “1”, the infecting and colonizing isolates were designated “101” and “102”, respectively.

### Bacterial isolates and CRE definition

Sample collection was performed using an anorectal swab method. Sterile, disposable transport medium (G1022-12; Gongdong Biotechnology, Zhejiang, China) was used to maintain specimen integrity. All samples were transported to the clinical microbiology laboratory at ambient temperature and processed within 2 h of collection to preserve microbial viability. CRE isolation was conducted by inoculating freshly collected anorectal swabs onto MacConkey agar. A meropenem disk (10 µg; Oxoid™, UK) was placed at the junction of the first and second quadrants following a standardized four-quadrant streaking technique. After 48 h of incubation at 35 °C in 5% CO₂, *Enterobacterales* isolates exhibiting an inhibition zone diameter of ≤ 22 mm (for 10 µg disks) around meropenem were preliminarily classified as putative CRE. Species identification was performed using the VITEK^®^ 2 automated system (bioMérieux, France). Antimicrobial susceptibility testing was conducted, and results were interpreted according to the Clinical and Laboratory Standards Institute guidelines (CLSI, 2023). Putative CRE isolates were confirmed for carbapenemase production using the modified carbapenem inactivation method (mCIM) and the EDTA-modified carbapenem inactivation method (eCIM), as per CLSI standards. Quality control was performed using *Escherichia coli* ATCC 25,922 and *Klebsiella pneumoniae* ATCC 700,603. All confirmed CRE isolates were stored at − 80 °C for subsequent analysis.

### Whole-genome sequencing and bioinformatic analysis

To characterize the pathogens responsible for secondary infections following CRE colonization, we performed WGS on paired colonizing and infecting isolates from 18 patients who developed subsequent infections after CRE colonization. Strain recovery failed for both the colonizing and infecting isolates from patient 14 and for the colonizing isolate from patient 18; therefore, WGS was not performed for these isolates. Ultimately, 33 CRE isolates—16 colonizing and 17 infecting—underwent successful WGS at Novogene Bioinformatics Institute (Beijing). Raw sequencing reads were quality-assessed using FastQC (v0.11.2). Adapters, low-quality bases, and reads with excessive ambiguous nucleotides were trimmed or filtered using fastx_toolkit to generate high-quality clean reads. *De novo* genome assembly was performed using SPAdes. For each genome assembled *de novo*, coding sequences (CDSs) were predicted with Prodigal v2.6.3 and subsequently annotated using Prokka v1.14.6. After annotation, the assembled genomes were screened against the Comprehensive Antibiotic Resistance Database (CARD, https://card.mcmaster.ca/) and the PubMLST database for multilocus sequence typing (MLST, https://cge.cbs.dtu.dk/services/MLST/). For paired colonization and secondary infection *K. pneumoniae* isolates, average nucleotide identity (ANI) was first calculated using FastANI v1.34, followed by analysis of virulence genes and associated virulence scores using Kleborate v3.1.3 with default parameters. A core-genome alignment was generated by identifying core genes across all isolates with Roary v3.13.0, using the -e -mafft parameters. Core-genome single nucleotide polymorphisms (cgSNPs) were extracted from this alignment using SNP-sites v2.5.1. Regions with recombinant signal were identified and excised using Gubbins v2.3.4. A maximum likelihood phylogenetic tree was then reconstructed from the recombination-filtered cgSNP alignment using RAxML v8.2.12 under the GTRGAMMA model, with branch support assessed via 1,000 bootstrap replicates [27]. The final tree was visualized with iTOL [28]. The raw WGS data generated in this study have been deposited in the NCBI Sequence Read Archive (SRA) under the BioProject accession number PRJNA1261240.

### Homology analysis

Genetic relatedness among strains was primarily assessed through WGS. As a complementary method, protein profile-based homology was evaluated using the MALDI-TOF MS system (bioMérieux, France) in Research Use Only (RUO) mode. Spectral profiles were acquired under 75 Hz laser irradiation across a mass-to-charge (m/z) range of 2,000–20,000 Da. Resulting spectra were comparatively analyzed using the SARAMIS software suite.

### Data collection and quality control

Clinical data were retrospectively extracted from the hospital information system (HIS), laboratory information system (LIS), and electronic medical records (EMR). The collected variables encompassed:

Demographics: age, sex, admitting department, and ICU length of stay.

Clinical characteristics: comorbidities, history of chemotherapy or radiotherapy, fecal incontinence, and presence of agranulocytosis (defined as an absolute neutrophil count < 500/µL).

Infection-related details: time of infection onset, infection sites, and causative CRE isolates.

Antibiotic exposure: types of antimicrobials administered, duration of therapy, and cumulative exposure.

Invasive procedures: use of urinary catheters, nasogastric tubes, endotracheal intubation, or tracheostomy.

During chart review, two independent clinicians assessed the clinical timeline and documentation to adjudicate whether a positive culture from a sterile site (e.g., blood) represented a primary infection or was secondary to a preceding non-sterile site infection (e.g., pneumonia or urinary tract infection). For this study, the first positive CRE culture associated with clinical signs of infection was considered the index infection event.

To ensure data accuracy and consistency, all extracted information underwent independent review by two investigators. Any discrepancies were resolved through consensus or by adjudication from a third senior researcher.

### Statistical analysis

All statistical analyses were performed using SPSSPRO (www.spsspro.com). Categorical variables, including demographic characteristics, clinical comorbidities, history of invasive procedures, and antibiotic exposures, were expressed as counts and percentages. Intergroup comparisons were conducted using two-tailed chi-square tests, with a *P*-value < 0.05 considered statistically significant. Variables showing significant associations in the univariate analysis were entered into a multivariable logistic regression model to identify independent risk factors for secondary infection. The discriminative ability of the resulting model was assessed using receiver operating characteristic (ROC) curve analysis, with the area under the curve (AUC) reported. Based on the final multivariable model, a nomogram for predicting the risk of secondary infection was constructed using R software. Calibration curves were plotted to evaluate the agreement between predicted probabilities and observed outcomes.

## Results

### CRE intestinal colonization and secondary infection rates

**Fig. 1:**
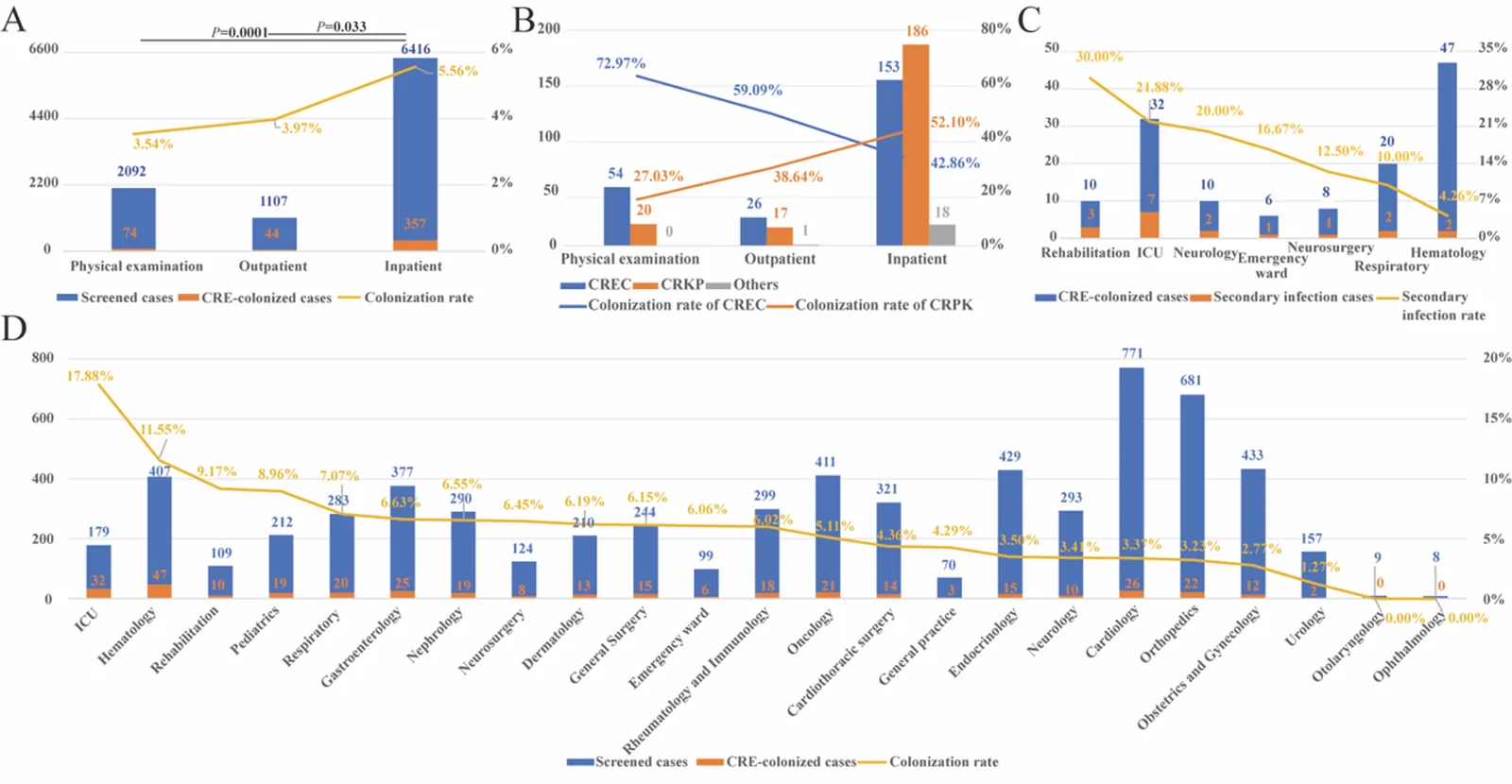
Intestinal colonization and secondary infection rates of CRE, and pathogen composition. **A** CRE intestinal colonization rates among individuals from the physical examination, outpatient, and inpatient groups. **B** Intestinal colonization rates of CREC and CRKP in the physical examination, outpatient, and inpatient populations. **C** Secondary infection rates of CRE across different hospital wards. **D** Intestinal colonization rates of CRE in various hospital wards. CRE, carbapenem-resistant *Enterobacterales*; CRKP, carbapenem-resistant *Klebsiella pneumoniae;* CREC, carbapenem-resistant *Escherichia coli*

Between August 31, 2021, and February 28, 2022, a total of 9,615 patients visiting our hospital underwent unbiased active screening for intestinal CRE carriage. Of these, 475 patients screened positive for intestinal CRE colonization. The colonization rate was 3.54% (74/2,092) in the physical examination group, 3.97% (44/1,107) in outpatients, and 5.56% (357/6,416) in inpatients. The colonization rates differed significantly across the physical examination, outpatient, and inpatient groups, with specific contrasts showing a significant difference between the physical examination and inpatients (*P* = 0.0001) and between outpatients and inpatients (*P* = 0.033) by Fisher’s exact test (Fig. 1A).

A distinct, inverse trend in species distribution was observed among the CRE carriers. While *Escherichia coli* (49.05%, 233/475) was overall more common than *Klebsiella pneumoniae* (46.95%, 223/475), the prevalence of carbapenem-resistant *Escherichia coli* (CREC) was highest in the physical examination group (72.97%) and decreased significantly in inpatients (42.86%). Conversely, carbapenem-resistant *Klebsiella pneumoniae* (CRKP) showed the opposite pattern, with its prevalence increasing from the physical examination group (27.03%) to the inpatient group (52.10%) (Fig. 1B).

The active screening identified 357 inpatients with intestinal CRE colonization. These colonized cases were widely distributed, covering 91.30% (21/23) of all hospital departments, with only the Otolaryngology and Ophthalmology departments showing no detected cases—a finding likely attributable to the relatively low number of screened cases in these two units. Analysis of colonization rates by department revealed that the top three departments with the highest rates were the ICU at 17.88% (32/179), the Hematology at 11.55% (47/407), and the Rehabilitation at 9.17% (10/109) (Fig. 1D).

During the 6-month follow-up period, 18 of the 357 colonized inpatients progressed to subsequent infection, resulting in an overall secondary infection rate of 5.04% (18/357). These infection cases were distributed across seven different departments, with notable inter-departmental variation in infection rates. Specifically, the highest secondary infection rates were observed in the Rehabilitation (30.00%, 3/10), the ICU (21.88%, 7/32), and the Neurology (20.00%, 2/10). Of note, although the Hematology had the largest number of CRE intestinal carriers (47 cases, accounting for 13.2% of all colonized inpatients), it exhibited the lowest secondary infection rate among all departments with infection cases, at only 4.26% (2/47) (Fig. 1C).

### Time from colonization to secondary infection

Among the 18 patients with secondary infections, the time from colonizing isolate detection to infecting isolate isolation ranged from 1 to 56 days (median: 8 days; mean: approximately 17 days). Most secondary infections were caused by isolates sharing the same sequence type (ST) as the colonizing isolates (e.g., 9 of 18 patients with ST11 *Kpn*), regardless of the time interval. Notably, in Patient 2 (time: 55 days), the infecting isolate (ST11 *Kpn*) differed from the colonizing isolate (ST609 *E. coli*), suggesting that longer intervals did not systematically favor heterologous infections. Multivariable Cox regression and logistic regression analyses, adjusting for age, underlying diseases, and antibiotic exposure, showed that time to event was not independently associated with the risk of secondary infection nor with strain homology (*P* > 0.05). Stratification by the median time (8 days) also revealed no significant differences in outcomes between groups. These findings indicate that time from colonization to infection was not a significant confounder in this study.

**Fig. 2:**
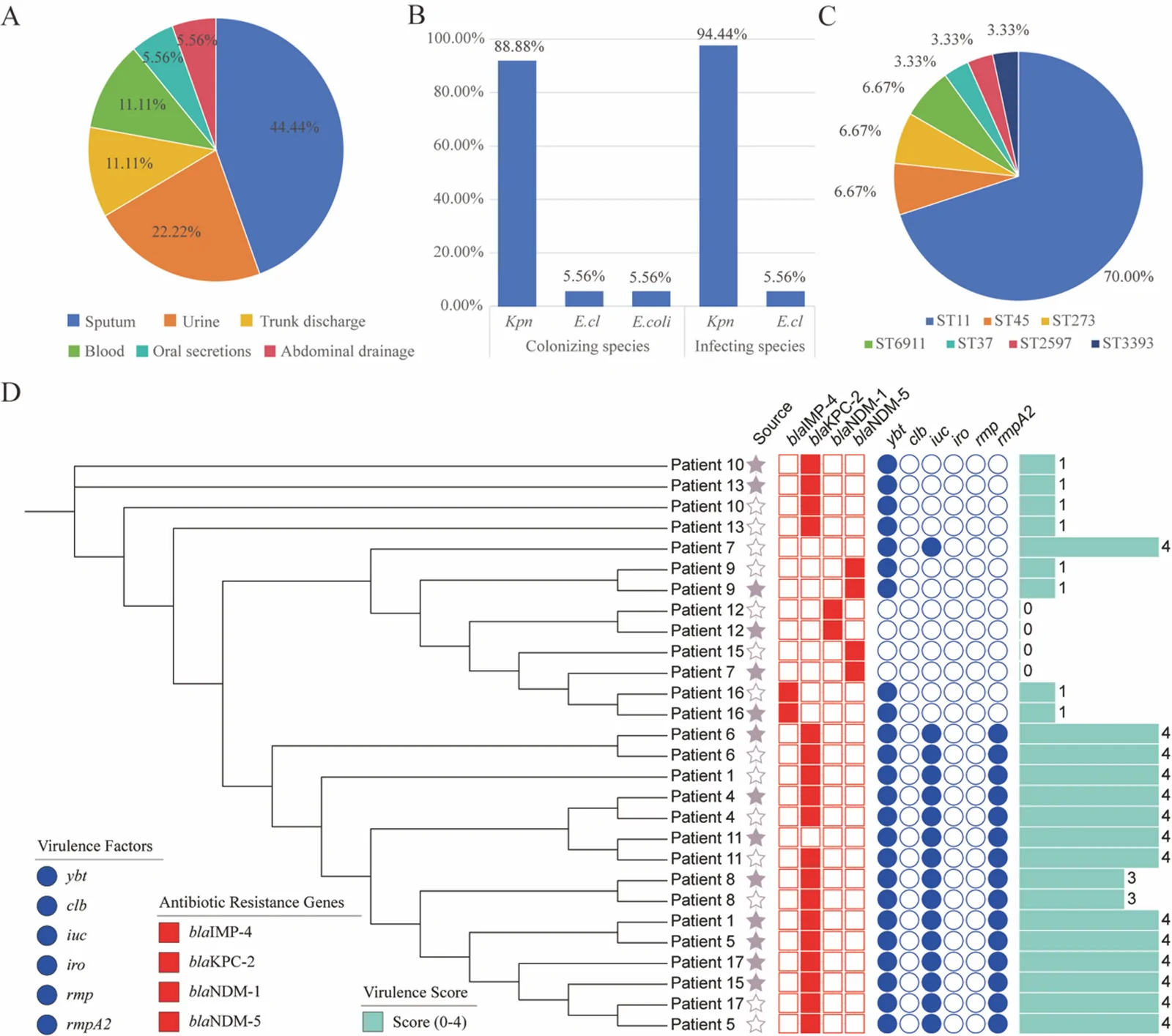
Microbiological and molecular characteristics of CRE colonization and subsequent infection. **A** Distribution of clinical specimen types from which secondary infection isolates were recovered. **B** Pathogen composition of the predominant bacterial species identified during initial CRE intestinal colonization and in subsequent infections. **C** The distribution of multilocus sequence typing (MLST) among CRKP isolates. **D** Genomic characteristics of *Klebsiella pneumoniae* isolates from colonized and infected patients. Isolation sources are indicated by star symbols: filled stars denote isolates from fecal samples, and open stars denote isolates from clinical infection sites. The presence or absence of key antibiotic resistance genes (ARGs) and virulence genes (VGs) is indicated by filled and open symbols, respectively. CRE, carbapenem-resistant *Enterobacterales*; CRKP, carbapenem-resistant *Klebsiella pneumoniae*

### Microbiological characteristics and genetic relatedness of CRE colonizing and secondary infection isolates

Sputum was the most common source of isolates from secondary infections (44.44%), followed by urine (22.22%), trunk discharge (11.11%), blood (11.11%), oral secretions (5.56%), and abdominal drainage (5.56%) (Fig. 2A). In terms of pathogen composition, both intestinal CRE colonization and secondary infections were predominantly caused by *Klebsiella pneumoniae*, accounting for 88.88% (16/18) and 94.44% (17/18) of cases, respectively (Fig. 2B). Exceptions included one patient (Patient 3) colonized and infected with *Enterobacter cloacae* (E.cl), and one patient (Patient 2) colonized with *Escherichia coli* but infected with *Klebsiella pneumoniae*. The remaining 16 patients exhibited secondary infections caused by *Klebsiella pneumoniae* following intestinal colonization (Table 1).

CRKP isolates were classified into 7 sequence types (STs): ST11, ST45, ST273, ST37, ST2597, ST3393, and ST6911, with ST11 being the dominant type, representing 70% (21/30) of the isolates (Fig. 2C). Additionally, two carbapenem-resistant *Enterobacter cloacae* (CR-E.cl) isolates were identified as ST156, and one CREC strain belonged to ST609 (Table 1).

**Fig. 3:**
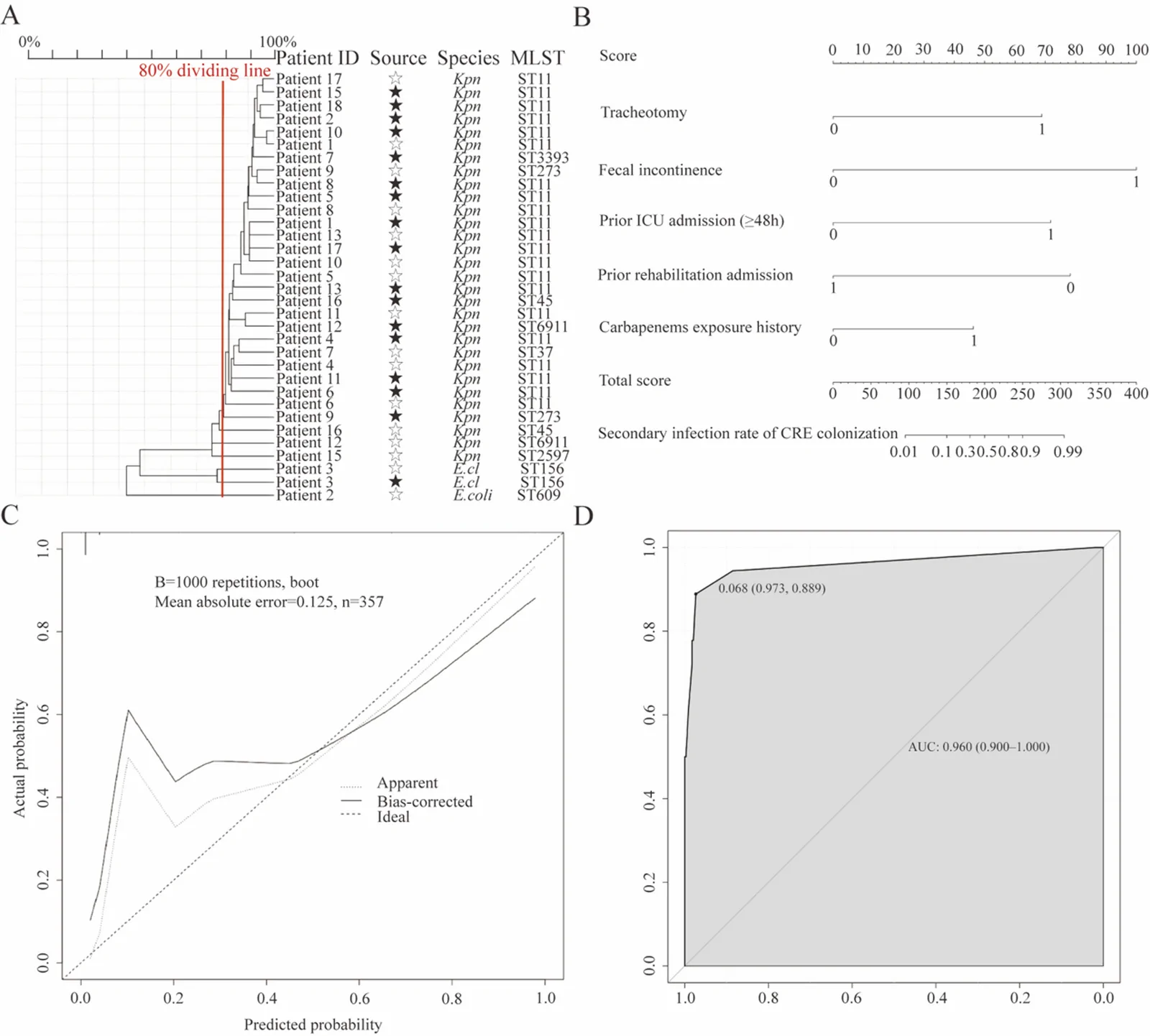
Homology analysis of colonizing and infecting CRE isolates and development of a risk prediction model for secondary infection. **A** Cluster analysis of MALDI-TOF MS protein spectra (bioMérieux) illustrating genetic relatedness among CRE strains isolated from intestinal colonization and subsequent clinical infection sites. Isolation sources are indicated by star symbols: filled stars denote isolates from fecal samples, and open stars denote isolates from clinical infection sites. **B** A clinically applicable nomogram for predicting the individual risk of secondary infection in patients with intestinal CRE colonization. The model incorporates five independent predictors identified by multivariate logistic regression. **C** Calibration curve of the nomogram, which plots the predicted probability of secondary infection against the observed frequency. The dashed diagonal line represents a perfect prediction, while the solid line shows the model’s performance. **D** Receiver operating characteristic (ROC) curve evaluating the discriminatory power of the multivariate logistic regression model. *Kpn*, *Klebsiella pneumoniae*; *E.coli*, *Escherichia coli*; *E.cl*, *Enterobacter cloacae*; MLST: multilocus sequence typing

To determine whether secondary infections originated from intestinal colonization, sequence types of colonizing and infecting isolates from the same patients were compared. Results showed that 13 patients had identical STs in their colonizing and infecting isolates, suggesting that the infections likely originated from the prior colonizing bacteria (Table 1). To further support this inference, MALDI-TOF MS-based cluster analysis was performed. Similarity among CRKP isolates of the same ST ranged from 76% to 97%, while the two CREC isolates showed 78% similarity. In contrast, similarity between different species was consistently below 50%, further supporting genetic homology among isolates sharing the same ST (Fig. 3A). To further validate the genetic relatedness at the whole-genome level, we calculated the average nucleotide identity (ANI) between paired colonization and secondary infection isolates from the same patients (Supplementary Table 1). The ANI values ranged from 98.9781% to 99.9985%, with the majority exceeding 99.97%. These results provide robust genomic evidence that the secondary infection isolates were nearly identical to the prior colonizing strains, strongly supporting a clonal origin [29].

Whole-genome sequencing further revealed the distribution of key antibiotic resistance genes (ARGs) and virulence genes (VGs) among colonizing and secondary infection isolates (Fig. 2D). Among the four carbapenemase genes examined, *bla*KPC-2 was the most prevalent, detected in 20/28 isolates (71.4%) and strongly associated with ST11. *bla*NDM-5 was present in four isolates, while *bla*NDM-1 occurred only in Patient 12 and *bla*IMP-4 exclusively in Patient 16. Regarding virulence genes, *ybt* was widely distributed (22/28 isolates, 78.6%), whereas *clb* and *iro* were completely absent. The hypervirulence-associated genes *iuc*, *rmp* and *rmpA2* were almost always co-present, restricted to isolates with a Virulence score ≥ 3 (12 isolates, all ST11 except Patient 7-infecting which belonged to ST3393). The siderophore receptor *iutA* was present in most high-score isolates but missing in a few (e.g., Patient 12-colonizing, Patient 17-infecting, Patient 4-colonizing).

When comparing colonizing and infection isolates from the same patient, most paired isolates (11/14) showed identical ARG and VG profiles and the same Virulence score, indicating stable genomic inheritance during transition from colonization to infection. However, two patients exhibited discordant patterns. In Patient 7, the colonizing isolate (Source 1) carried only *bla*NDM-5 (score 0), whereas the secondary infection isolate (Source 2) harbored *ybt*, *iuc* and *rmp* (score 4) but no carbapenemase gene, suggesting acquisition of a hypervirulent clone or loss of the carbapenemase during infection. In Patient 15, the colonizing isolate possessed *bla*KPC-2 together with *iuc*, *rmp* and *rmpA2* (score 4), while the infection isolate carried only *bla*NDM-5 (score 0), indicating either co-colonization with distinct strains or replacement of the colonizing strain by a different infecting clone. Overall, high Virulence scores (≥ 3) were almost exclusively observed in ST11 isolates carrying *bla*KPC-2, except for one ST3393 infection isolate (Patient 7-infecting) that achieved a score of 4 without any carbapenemase gene.

In summary, evidence from pathogen distribution, molecular typing, genetic similarity, and genomic profiling consistently demonstrated that secondary infections were primarily caused by *Klebsiella pneumoniae*, with ST11 being the dominant epidemic type. Most infection events showed high genetic relatedness to the intestinally colonizing isolates at the molecular level. The co-occurrence of *bla*KPC-2 and hypervirulence genes (*iuc*, *rmp*, *rmpA2*) was characteristic of the majority of secondary infection isolates.

**Table 1:** Detailed characteristics of colonizing and infecting isolates in patients with secondary infections following CRE intestinal colonization

Patient ID	Isolation date of colonizing isolates	Colonizing isolates	MLST of colonizing isolates	Isolation date of infecting isolates	Infecting isolates	Types of specimens	MLST of infecting isolates	Time to event
Patient 1	2021-10-22	*Kpn*	ST11	2021-11-30	*Kpn*	Urine	ST11	39
Patient 2	2021-09-10	*E.coli*	ST609	2021-11-04	*Kpn*	Sputum	ST11	55
Patient 3	2021-12-12	*E.cl*	ST156	2022-01-11	*E.cl*	Sputum	ST156	30
Patient 4	2021-09-28	*Kpn*	ST11	2021-10-19	*Kpn*	Sputum	ST11	21
Patient 5	2021-09-16	*Kpn*	ST11	2021-09-24	*Kpn*	Urine	ST11	8
Patient 6	2021-09-12	*Kpn*	ST11	2021-09-18	*Kpn*	Blood	ST11	6
Patient 7	2021-11-27	*Kpn*	ST37	2021-12-08	*Kpn*	Sputum	ST3393	11
Patient 8	2021-09-02	*Kpn*	ST11	2021-09-03	*Kpn*	Urine	ST11	1
Patient 9	2021-11-23	*Kpn*	ST273	2021-11-26	*Kpn*	Blood	ST273	3
Patient 10	2021-12-14	*Kpn*	ST11	2021-12-17	*Kpn*	Sputum	ST11	3
Patient 11	2021-09-18	*Kpn*	ST11	2021-10-29	*Kpn*	Trunk discharge	ST11	41
Patient 12	2021-09-10	*Kpn*	ST6911	2021-09-15	*Kpn*	Oral secretions	ST6911	5
Patient 13	2021-11-06	*Kpn*	ST11	2021-11-09	*Kpn*	Sputum	ST11	3
Patient 14	2021-12-11	*Kpn*	-	2021-12-16	*Kpn*	Abdominal drainage	-	5
Patient 15	2021-10-28	*Kpn*	ST2597	2021-11-05	*Kpn*	Urine	ST11	8
Patient 16	2021-09-13	*Kpn*	ST45	2021-09-22	*Kpn*	Trunk discharge	ST45	9
Patient 17	2021-09-03	*Kpn*	ST11	2021-10-29	*Kpn*	Sputum	ST11	56
Patient 18	2021-11-21	*Kpn*	-	2021-11-23	*Kpn*	Sputum	ST11	2

“-“means the strain failed resuscitation and excluded in the WGS and homology analysis. Abbreviations: *Kpn*, *Klebsiella pneumoniae*; *E.coli*, *Escherichia coli*; *E.cl*, *Enterobacter cloacae*; MLST: multilocus sequence typing

### Predicting secondary infection following CRE colonization: A risk factor analysis and model development

Based on follow-up medical records, the 357 inpatients with intestinal CRE colonization were categorized into two groups: “Secondary infection group” (*n* = 18) and “Colonized uninfected group” (*n* = 339). Univariate analysis revealed multiple significant differences (*P* < 0.05) in clinical characteristics, invasive procedures, and antibiotic exposure between the two groups. Specifically, fecal incontinence, admission to Rehabilitation, and ICU stay ≥ 48 h were significantly associated clinical factors. In terms of invasive procedures, the use of long-term indwelling urinary catheter, endotracheal intubation, indwelling gastrojejunal tube, and tracheostomy was significantly more frequent in the infection group. Additionally, patients in the infection group had greater exposure to β-lactam/β-lactamase inhibitor combinations, fluoroquinolones, carbapenems, tetracyclines, aminoglycosides, and oxazolidinones after colonization (Table 2).

**Table 2:** Univariate analysis of secondary infections in patients with intestinal CRE colonization

Demographics and epidemiological context	Secondary infection group (*n* = 18)	Colonized uninfected group (*n* = 339)	X^2^ value	*P*
Age ≥ 65	7	82	3.709	0.054
Male sex	8	261	1.131	0.287
Diabetes	5	50	1.339	0.247
Granulocytopenia	2	14	-	0.149
History of chemoradiotherapy	3	73	0.038	0.845
Immunosuppressive therapy	1	16	-	0.594
Fecal incontinence	10	5	-	0.000
ICU stay ≥ 48 h	11	5	-	0.000
Admission to Rehabilitation	6	10	-	0.000
Long-term indwelling urinary catheter	14	13	123.309	0.000
Postoperative indwelling urinary catheter	0	56	2.388	0.122
Endotracheal intubation	4	4	-	0.000
Indwelling gastrojejunal tube	14	22	88.099	0.000
Tracheotomy	11	6	-	0.000
β-lactam/β-lactamase inhibitor combinations	12	67	19.183	0.000
Fluoroquinolones	4	22	4.152	0.042
Folate pathway antagonists	2	28	0	1.000
Cephalosporins	3	79	0.133	0.715
Carbapenems	15	35	69.705	0.000
Tetracyclines	6	10	-	0.000
Aminoglycosides	4	4	-	0.000
Polypeptides	2	13	-	0.171
Oxazolidinones	4	3	-	0.000

CRE, carbapenem-resistant *Enterobacteriaceae;* ICU, Intensive Care Unit

To identify independent risk factors for subsequent infection, variables with statistical significance in the univariate analysis were incorporated into a multivariable logistic regression model. The results demonstrated that ICU stay ≥ 48 h, prior exposure to carbapenems, fecal incontinence, and tracheostomy were independent risk factors for the progression from CRE colonization to clinical infection. In contrast, admission to Rehabilitation was identified as a protective factor (Table 3).

**Table 3:** Multivariate logistic analysis of secondary infections in patients with intestinal CRE colonization

Risk factors	B value	Significance	Exp (B)	95%CI
Tracheotomy	5.509	0.027	157.5	1.753-14152.521
Admission to Rehabilitation	-6.926	0.045	0.001	0-0.848
ICU stay ≥ 48 h	4.214	0.01	67.615	2.791-1637.776
Fecal incontinence	6.147	0.013	467.383	3.741-58391.517
Carbapenems	2.662	0.022	14.32	1.464-140.031

ICU, Intensive Care Unit

Based on these five independent factors—tracheostomy, fecal incontinence, ICU stay ≥ 48 h, admission to Rehabilitation, and carbapenem exposure—a nomogram model was constructed to individually predict the risk of secondary infection in patients with intestinal CRE colonization (Fig. 3B). The calibration curve indicated moderate agreement between predicted and observed outcomes in the low-risk group (predicted probability < 50%), while excellent agreement was observed in the high-risk group (predicted probability ≥ 50%), suggesting superior clinical applicability for identifying high-risk patients (Fig. 3C).

The predictive performance of the logistic regression model was further evaluated using the receiver operating characteristic (ROC) curve, which yielded an area under the curve (AUC) of 0.960 (95% CI: 0.900–1.000). The Youden index was 0.862, with model sensitivity and specificity of 88.9% and 97.3%, respectively, indicating excellent discriminatory ability and high predictive accuracy (Fig. 3D).

## Discussion

Monitoring intestinal colonization of CRE in healthcare settings is critical for understanding intra- and inter-hospital transmission chains. This study revealed an overall CRE carriage rate of 3.54%–5.55% in fecal samples from a tertiary general hospital, with significant variation among different populations: inpatients exhibited a higher carriage rate (5.55%) than outpatients (3.97%) and individuals undergoing health examinations (3.54%). This discrepancy may be attributed to inpatients’ more frequent exposure to the healthcare environment, invasive procedures, and antimicrobial agents. A children’s hospital in Shanghai previously reported a pediatric intestinal CRE carriage rate of 3.6% [30], while Chen et al. [31] documented a rapid increase in CRE carriage among rural healthy residents in Shandong province, from 2.4% in 2015 to 13.4% in 2017. Together, these findings indicate that CRE colonization is not limited to inpatients but also involves outpatients and healthy individuals in contact with healthcare facilities, underscoring the need for strengthened infection prevention and control.

At the departmental level, high CRE colonization rates (7.07%–17.88%) were observed in the ICU, hematology, rehabilitation, pediatrics, and respiratory departments. Colonization rates in the ICU (17.88% vs. 18.58%) and hematology ward (11.55% vs. 10.81%) were comparable to those reported in another Chinese hospital [8]. The pediatric ward colonization rate in this study (8.96%) was higher than rates reported in Guangzhou (3.92%) and Fujian (6.6%), but similar to those in Hunan (8.5%) and Shanghai (8.6%) [32–34]. Meanwhile, the high CRE colonization rate in the rehabilitation ward (9.17%) was lower than that reported in an Italian hospital (11.6%) [35]. Patients in these high-risk wards often present with immunosuppression, broad-spectrum antibiotic exposure, and frequent invasive procedures. In terms of pathogen distribution, *Escherichia coli* and *Klebsiella pneumoniae* were the predominant CRE species, consistent with previous studies [10]. Notably, our findings underscore the distinct epidemiological profiles of CRKP and CREC: CRKP demonstrates a clear hospital-acquired pattern, with colonization concentrated among inpatients and posing a outbreak risk predominantly in clinical wards. Conversely, CREC is widely distributed across various healthcare settings. This finding holds practical value for informing tailored infection prevention, control, and focused surveillance strategies across different clinical departments.

Regarding risk factors for secondary infection, this study identified ICU stay ≥ 48 h, carbapenem exposure, fecal incontinence, and tracheostomy as independent risk factors, while admission to Rehabilitation was a protective factor. A predictive model constructed using these variables demonstrated excellent discriminative ability (AUC = 0.960). While the nomogram exhibited high discriminative performance within our cohort, it is crucial to interpret this as an initial step in risk stratification. External validation in larger, multi-center cohorts is essential before this tool can be applied in routine clinical practice. And a nomogram was developed to facilitate individualized risk assessment. Fecal incontinence may increase the risk of CRE translocation and infection through perineal contamination and impaired skin barrier integrity. Prolonged ICU stay is often accompanied by critical illness, multiple invasive procedures, and antibiotic pressure, collectively contributing to gut microbiota disruption and pathogen dissemination. Patients in rehabilitation departments generally have more stable clinical conditions, require fewer invasive interventions, and experience lower risks of cross-transmission, which may explain the protective effect observed.

Furthermore, antibiotic exposure was confirmed as a key driver of the transition from colonization to infection. The use of β-lactam/β-lactamase inhibitor combinations, fluoroquinolones, carbapenems, and other drug classes was significantly associated with subsequent infection. The underlying mechanisms may involve two aspects: first, broad-spectrum antibiotics disrupt gut microbiota and compromise colonization resistance; second, sustained drug pressure selects for resistant clones and facilitates their expansion and transmission. Therefore, implementing precise antimicrobial stewardship in CRE-colonized patients is essential.

From a molecular epidemiological perspective, ST11 CRKP was the dominant clone in both colonization and infection isolates (70%), and most patients’ colonization and infection isolates were genetically homologous, supporting the notion of nosocomial transmission and endogenous infection. Previous studies have indicated that the ST11 clone carries multiple resistance islands (e.g., ramR-lon, pmrB-phoQ-mgrB), which may mediate resistance to tigecycline, colistin, and other agents [36, 37]. This could partly explain its strong colonization persistence and heightened potential to cause infection.

Extensive prior work has established the gastrointestinal tract as the primary reservoir for *Klebsiella pneumoniae* infection, with colonized patients facing a significantly elevated risk of subsequent infection (odds ratios ranging from 4.0 to 6.9) and the infecting strain being genetically concordant with the antecedent colonizing isolate in the majority of cases [38–41]. Progression from colonization to infection is modulated not only by patient comorbidities and hypoalbuminemia [41, 42] but also by specific microbial genomic features, including plasmid-borne antimicrobial resistance and stress-adaptation genes that enhance gut fitness [43, 44]. Furthermore, integrated genomic and patient-transfer network analyses have demonstrated that interfacility patient movement is a dominant driver of regional CRKP dissemination, often linking acute care hospitals with long-term care facilities [45, 46]. These foundational findings align with our observation of ST11 clonal dominance and endogenous infection patterns, reinforcing the critical need for both targeted surveillance of high-risk colonized patients and coordinated infection control across the healthcare continuum.

This study has several limitations. First, although we conducted a large-scale, untargeted active surveillance program within our hospital, the single-center design may still introduce selection bias and limit the generalizability of our findings to broader populations. Second, due to greater challenges in following outpatients and individuals undergoing routine health examinations compared to inpatients, only hospitalized patients were included in the six-month follow-up. Consequently, only 18 cases of post-colonization infection were recorded, resulting in a relatively small sample size for analyzing infection outcomes. Third, although the nomogram prediction model demonstrated excellent discriminative ability, its clinical utility requires further validation through external multicentre cohorts.

## Conclusion

In summary, this study systematically delineated the epidemiological profile of intestinal CRE colonization, identified risk factors for subsequent infection and their molecular underpinnings, and developed a well-performing predictive tool. The findings emphasize the need to enhance active screening and intervention in high-risk wards and populations, optimize antibiotic use, and strengthen surveillance of dominant clones such as ST11 CRKP, thereby effectively curbing CRE transmission and infection occurrence.

## Supplementary Information

Below is the link to the electronic supplementary material.

Supplementary Material 1.

## Data Availability

The whole-genome sequencing datasets produced in this work are accessible in the NCBI Sequence Read Archive (SRA) under the accession number PRJNA1261240. Correspondence and requests for other raw datasets should be addressed to Zhaofan Luo at [luozhaofan@sysush.com](mailto: luozhaofan@sysush.com) .
