# Supplementary material for: A nomogram for assessing secondary infection risk in hospitalized patients with intestinal CRE colonization: a prospective cohort study
Source: Ann Clin Microbiol Antimicrob. 2026 May 29;25:31. doi: 10.1186/s12941-026-00867-2 (PMC13491868; doi:10.1186/s12941-026-00867-2)
Supplement: Supplementary file 1 [file 12941_2026_867_MOESM1_ESM.docx]

Supplementary Table 1.  The average nucleotide identity (ANI) between paired colonization and secondary infection isolates from the same patients

| Secondary infection isolate | Colonization isolate | ANI |
| --- | --- | --- |
| Patient 10 | Patient 10 | 99.9971 |
| Patient 11 | Patient 11 | 99.9721 |
| Patient 12 | Patient 12 | 99.9967 |
| Patient 13 | Patient 13 | 99.9959 |
| Patient 15 | Patient 15 | 98.9781 |
| Patient 16 | Patient 16 | 99.9983 |
| Patient 17 | Patient 17 | 99.9958 |
| Patient 1 | Patient 1 | 99.9794 |
| Patient 4 | Patient 4 | 99.9966 |
| Patient 5 | Patient 5 | 99.9971 |
| Patient 6 | Patient 6 | 99.9959 |
| Patient 7 | Patient 7 | 99.1093 |
| Patient 8 | Patient 8 | 99.9939 |
| Patient 9 | Patient 9 | 99.9985 |
